# ITC-Net-blend-60: a comprehensive dataset for robust network traffic classification in diverse environments

**DOI:** 10.1186/s13104-024-06817-5

**Published:** 2024-06-15

**Authors:** Marziyeh Bayat, Javad Garshasbi, Mozhgan Mehdizadeh, Neda Nozari, Abolghasem Rezaei Khesal, Maryam Dokhaei, Mehdi Teimouri

**Affiliations:** Information Theory and Coding (ITC) Laboratory, University of Tehran, Tehran, Iran

**Keywords:** Network traffic analysis, Traffic classification, Application identification, Mobile-app fingerprinting, Encrypted traffic, Android applications, Robustness, Various network environments, Raw labeled dataset

## Abstract

**Objectives:**

Recognition of mobile applications within encrypted network traffic holds considerable effects across multiple domains, encompassing network administration, security, and digital marketing. The creation of network traffic classifiers capable of adjusting to dynamic and unforeseeable real-world settings presents a tremendous challenge. Presently available datasets exclusively encompass traffic data obtained from a singular network environment, thereby restricting their utility in evaluating the robustness and compatibility of a given model.

**Data description:**

This dataset was gathered from 60 popular Android applications in five different network scenarios, with the intention of overcoming the limitations of previous datasets. The scenarios were the same in the applications set but differed in terms of Internet service provider (ISP), geographic location, device, application version, and individual users. The traffic was generated through real human interactions on physical devices for 3–15 min. The method used to capture the traffic did not require root privileges on mobile phones and filtered out any background traffic. In total, the collected dataset comprises over 48 million packets, 450K bidirectional flows, and 36 GB of data.

**Supplementary Information:**

The online version contains supplementary material available at 10.1186/s13104-024-06817-5.

## Objective

As the volume of mobile app traffic continues to soar, the importance of reliable app identification solutions cannot be understated. Mobile app traffic identification aids network management and security and provides valuable profiling information for advertisers, insurance companies, and security agencies [12–14]. As a result, it has garnered significant interest from both academia and industry, leading to extensive research on the subject.

However, achieving robust application identification remains an open problem. Recent investigations [15, 16] have revealed that although existing classifiers achieve satisfactory performance when trained and tested using conventional machine learning methods (dividing a dataset into two parts for training and testing), most of them face significant performance degradation when evaluated with different datasets. This indicates a lack of robustness and compatibility of models in practical networks. This challenge stems from the unpredictable nature of real-world network environments [15, 16] and the dynamic and evolving behavior of mobile apps [13, 17–19]. A key requirement for achieving this goal is a dataset of captured traffic data in various network scenarios. Nonetheless, as indicated in S Table 3, existing datasets were mainly captured in a single invariant network environment.

In this paper, we address this limitation by presenting a dataset that was captured across five different network scenarios, with various factors affecting network traffic behavior. This dataset allows for the evaluation of model performance under different network conditions. It was provided in raw format (PCAP files) to give researchers the flexibility to develop models based on any traffic object (e.g., packet, flow, and bag of flows), feature, method, or innovative approaches. Moreover, this dataset was generated through real human interactions on actual smartphones and captured using a non-rooted method. This makes the data more representative and suitable than synthetic data for mobile app traffic analysis.

### Data description

The methodology employed for collecting the dataset comprised three main stages: Application Selection, Traffic Capture Setup, and Traffic Generation. Moore's details about each phase are provided in the accompanying supplementary materials.

### Application selection

To collect traffic data, an initial step is to determine which applications to monitor, given the vast number of applications available. We chose 60 Android applications from the top 300 free apps listed in the Google Play Store and two major Iranian Android app markets, Cafe Bazaar [1], and Myket [2]. Our selection was based on two criteria: the apps must require internet connectivity to fulfill their core functions, and they must generate traffic through user interactions. These 60 apps belong to 16 distinct categories, which are listed in S Table A1.

### Traffic capture setup

We used a smartphone and a laptop to capture our traffic. The laptop ran Windows 10 and had an internal dual-band network card. We installed Wireshark [3] on it and configured it to capture traffic through the “Local Area Connection” interface. The laptop was connected to the internet and shared its connection with the smartphone via a hotspot. This allowed Wireshark to capture the smartphone’s network traffic. However, the traffic captured by Wireshark also contained significant background traffic. To isolate the target application’s network traffic, we installed PCAPdroid [4] on the smartphone in non-root mode. We used Wireshark and PCAPdroid simultaneously to record the target application’s traffic. After collecting traffic data, we separated the target application traffic from the background traffic by comparing IP addresses and ports captured by Wireshark and PCAPdroid. Any pairs in Wireshark that did not match a PCAPdroid pair were identified as background traffic and removed.

We implemented this method in Python 3 using the Scapy library [5]. You can find the code for this implementation in the Supplementary material.

### Traffic generation

The dataset collection was conducted by five volunteers from ITC-LAB members over a period of six weeks, from October to December 2021. Each volunteer collected traffic from a different network Scenario (see S Table 1). Before commencing the data collection process, they were well-informed about the objectives of traffic capture and the public release of data in PCAP format. They also received training on how to collect traffic. The volunteers were required to conduct at least three experiments for every application, with each experiment consisting of interacting with a single app on a specific smartphone for 3 to 15 min. They were instructed to use the application as they normally would.

The resulting dataset is organized into separate repositories for each scenario, with a dedicated compressed file for every application. Each compressed file contains the corresponding PCAP files, all of which have been named using a consistent naming convention. The format for naming the PCAP files is as follows: (Application Name)_(Scenario ID)_(#Trace)_Final.pcap.

The entire dataset comprises 1,159 PCAP traces and 36 GB of network traffic data. S Table A2 provides further details about the dataset for each app.

### Limitations

- This dataset only includes traffic of a specific subset of Android applications. It does not include traffic from other operating systems or applications.
- Each capture session, represented by a PCAP file, has a fixed duration ranging from 3 to 15 min.
- Due to internet access restrictions in Iran, the traffic of ten applications—Coursera, Facebook Lite, Goodreads, Likee, Snapchat, Spotify, Telegram, Twitter, YouTube, and Waze—has been recorded differently compared to the other applications. (For more information, please refer to the detailed description provided in the Supplementary Materials.)

### Supplementary Information

Additional file1 (DOCX 370 KB)

## Data Availability

The data described in this Data note can be freely and openly accessed on Mendeley Data under the name ITC-Net-Blend-60. Please see Table

**Table 1:** Overview of data files/data sets

Label	Name of data set	File types (file extension)	Data repository and identifier (DOI or accession number)
Data file 1	Adobe Connect	Archive file format (.rar) containing PCAP files	Mendeley Data (https://doi.org/10.17632/ssv23kfcgs.3 [6])
Data file 2	Aparat	Archive file format (.rar) containing PCAP files	Mendeley Data (https://doi.org/10.17632/ssv23kfcgs.3 [6])
Data file 3	AP—Asan Pardakht	Archive file format (.rar) containing PCAP files	Mendeley Data (https://doi.org/10.17632/ssv23kfcgs.3 [6])
Data file 4	Balad	Archive file format (.rar) containing PCAP files	Mendeley Data (https://doi.org/10.17632/ssv23kfcgs.3 [6])
Data file 5	Bazaar	Archive file format (.rar) containing PCAP files	Mendeley Data (https://doi.org/10.17632/ssv23kfcgs.3 [6])
Data file 6	Castbox	Archive file format (.rar) containing PCAP files	Mendeley Data (https://doi.org/10.17632/ssv23kfcgs.3 [6])
Data file 7	Clash of Clans	Archive file format (.rar) containing PCAP files	Mendeley Data (https://doi.org/10.17632/ssv23kfcgs.3 [6])
Data file 8	Clubhouse	Archive file format (.rar) containing PCAP files	Mendeley Data (https://doi.org/10.17632/ssv23kfcgs.3 [6])
Data file 9	Coursera	Archive file format (.rar) containing PCAP files	Mendeley Data (https://doi.org/10.17632/ssv23kfcgs.3 [6])
Data file 10	Digikala	Archive file format (.rar) containing PCAP files	Mendeley Data (https://doi.org/10.17632/ssv23kfcgs.3 [6])
Data file 11	Discord	Archive file format (.rar) containing PCAP files	Mendeley Data (https://doi.org/10.17632/ssv23kfcgs.3 [6])
Data file 12	Divar	Archive file format (.rar) containing PCAP files	Mendeley Data (https://doi.org/10.17632/ssv23kfcgs.3 [6])
Data file 13	Dropbox	Archive file format (.rar) containing PCAP files	Mendeley Data (https://doi.org/10.17632/ssv23kfcgs.3 [6])
Data file 14	Duolingo	Archive file format (.rar) containing PCAP files	Mendeley Data (https://doi.org/10.17632/ssv23kfcgs.3 [6])
Data file 15	Eitaa	Archive file format (.rar) containing PCAP files	Mendeley Data (https://doi.org/10.17632/ssv23kfcgs.3 [6])
Data file 16	Ewano	Archive file format (.rar) containing PCAP files	Mendeley Data (https://doi.org/10.17632/ssv23kfcgs.3 [6])
Data file 17	Facebook Lite	Archive file format (.rar) containing PCAP files	Mendeley Data (https://doi.org/10.17632/ssv23kfcgs.3 [6])
Data file 18	Facelab	Archive file format (.rar) containing PCAP files	Mendeley Data (https://doi.org/10.17632/ssv23kfcgs.3 [6])
Data file 19	Fidibo	Archive file format (.rar) containing PCAP files	Mendeley Data (https://doi.org/10.17632/ssv23kfcgs.3 [6])
Data file 20	Filimo	Archive file format (.rar) containing PCAP files	Mendeley Data (https://doi.org/10.17632/ssv23kfcgs.3 [6])
Data file 21	Firefox Browser	Archive file format (.rar) containing PCAP files	Mendeley Data (https://doi.org/10.17632/ssv23kfcgs.3 [6])
Data file 22	Football Strike	Archive file format (.rar) containing PCAP files	Mendeley Data (https://doi.org/10.17632/ssv23kfcgs.3 [6])
Data file 23	Gmail	Archive file format (.rar) containing PCAP files	Mendeley Data (https://doi.org/10.17632/ssv23kfcgs.3 [6])
Data file 24	Goodreads	Archive file format (.rar) containing PCAP files	Mendeley Data (https://doi.org/10.17632/ssv23kfcgs.3 [6])
Data file 25	Google Chrome	Archive file format (.rar) containing PCAP files	Mendeley Data (https://doi.org/10.17632/ssv23kfcgs.3 [6])
Data file 26	Google Maps	Archive file format (.rar) containing PCAP files	Mendeley Data (https://doi.org/10.17632/ssv23kfcgs.3 [6])
Data file 27	Google Meet	Archive file format (.rar) containing PCAP files	Mendeley Data (https://doi.org/10.17632/ssv23kfcgs.3 [6])
Data file 28	Google Play Store	Archive file format (.rar) containing PCAP files	Mendeley Data (https://doi.org/10.17632/ssv23kfcgs.3 [6])
Data file 29	iGap	Archive file format (.rar) containing PCAP files	Mendeley Data (https://doi.org/10.17632/ssv23kfcgs.3 [6])
Data file 30	InSave	Archive file format (.rar) containing PCAP files	Mendeley Data (https://doi.org/10.17632/ssv23kfcgs.3 [6])
Data file 31	Instagram	Archive file format (.rar) containing PCAP files	Mendeley Data (https://doi.org/10.17632/ssv23kfcgs.3 [6])
Data file 32	Likee	Archive file format (.rar) containing PCAP files	Mendeley Data (https://doi.org/10.17632/ssv23kfcgs.3 [6])
Data file 33	LinkedIn	Archive file format (.rar) containing PCAP files	Mendeley Data (https://doi.org/10.17632/ssv23kfcgs.3 [6])
Data file 34	Mafioso	Archive file format (.rar) containing PCAP files	Mendeley Data (https://doi.org/10.17632/ssv23kfcgs.3 [6])
Data file 35	Memrise	Archive file format (.rar) containing PCAP files	Mendeley Data (https://doi.org/10.17632/ssv23kfcgs.3 [6])
Data file 36	Mencherz	Archive file format (.rar) containing PCAP files	Mendeley Data (https://doi.org/10.17632/ssv23kfcgs.3 [6])
Data file 37	Microsoft Outlook	Archive file format (.rar) containing PCAP files	Mendeley Data (https://doi.org/10.17632/ssv23kfcgs.3 [6])
Data file 38	Myket	Archive file format (.rar) containing PCAP files	Mendeley Data (https://doi.org/10.17632/ssv23kfcgs.3 [6])
Data file 39	Neshan	Archive file format (.rar) containing PCAP files	Mendeley Data (https://doi.org/10.17632/ssv23kfcgs.3 [6])
Data file 40	OneDrive	Archive file format (.rar) containing PCAP files	Mendeley Data (https://doi.org/10.17632/ssv23kfcgs.3 [6])
Data file 41	Pinterest	Archive file format (.rar) containing PCAP files	Mendeley Data (https://doi.org/10.17632/ssv23kfcgs.3 [6])
Data file 42	Quiz Of Kings	Archive file format (.rar) containing PCAP files	Mendeley Data (https://doi.org/10.17632/ssv23kfcgs.3 [6])
Data file 43	Radio Javan	Archive file format (.rar) containing PCAP files	Mendeley Data (https://doi.org/10.17632/ssv23kfcgs.3 [6])
Data file 44	Shaypur	Archive file format (.rar) containing PCAP files	Mendeley Data (https://doi.org/10.17632/ssv23kfcgs.3 [6])
Data file 45	Skype	Archive file format (.rar) containing PCAP files	Mendeley Data (https://doi.org/10.17632/ssv23kfcgs.3 [6])
Data file 46	Snapchat	Archive file format (.rar) containing PCAP files	Mendeley Data (https://doi.org/10.17632/ssv23kfcgs.3 [6])
Data file 47	Snapp	Archive file format (.rar) containing PCAP files	Mendeley Data (https://doi.org/10.17632/ssv23kfcgs.3 [6])
Data file 48	Soroush Plus Messenger	Archive file format (.rar) containing PCAP files	Mendeley Data (https://doi.org/10.17632/ssv23kfcgs.3 [6])
Data file 49	Spotify	Archive file format (.rar) containing PCAP files	Mendeley Data (https://doi.org/10.17632/ssv23kfcgs.3 [6])
Data file 50	Taghche	Archive file format (.rar) containing PCAP files	Mendeley Data (https://doi.org/10.17632/ssv23kfcgs.3 [6])
Data file 51	Tapsi	Archive file format (.rar) containing PCAP files	Mendeley Data (https://doi.org/10.17632/ssv23kfcgs.3 [6])
Data file 52	Telegram	Archive file format (.rar) containing PCAP files	Mendeley Data (https://doi.org/10.17632/ssv23kfcgs.3 [6])
Data file 53	Telewebion	Archive file format (.rar) containing PCAP files	Mendeley Data (https://doi.org/10.17632/ssv23kfcgs.3 [6])
Data file 54	ToonMe	Archive file format (.rar) containing PCAP files	Mendeley Data (https://doi.org/10.17632/ssv23kfcgs.3 [6])
Data file 55	Torob	Archive file format (.rar) containing PCAP files	Mendeley Data (https://doi.org/10.17632/ssv23kfcgs.3 [6])
Data file 56	Twitter	Archive file format (.rar) containing PCAP files	Mendeley Data (https://doi.org/10.17632/ssv23kfcgs.3 [6])
Data file 57	Waze	Archive file format (.rar) containing PCAP files	Mendeley Data (https://doi.org/10.17632/ssv23kfcgs.3 [6])
Data file 58	Whatsapp Messenger	Archive file format (.rar) containing PCAP files	Mendeley Data (https://doi.org/10.17632/ssv23kfcgs.3 [6])
Data file 59	Youtube	Archive file format (.rar) containing PCAP files	Mendeley Data (https://doi.org/10.17632/ssv23kfcgs.3 [6])
Data file 60	Adobe Connect	Archive file format (.rar) containing PCAP files	Mendeley Data (https://doi.org/10.17632/3zggb53m4x.3 [7])
Data file 61	AP—Asan Pardakht	Archive file format (.rar) containing PCAP files	Mendeley Data (https://doi.org/10.17632/3zggb53m4x.3 [7])
Data file 62	Aparat	Archive file format (.rar) containing PCAP files	Mendeley Data (https://doi.org/10.17632/3zggb53m4x.3 [7])
Data file 63	Balad	Archive file format (.rar) containing PCAP files	Mendeley Data (https://doi.org/10.17632/3zggb53m4x.3 [7])
Data file 64	Bazaar	Archive file format (.rar) containing PCAP files	Mendeley Data (https://doi.org/10.17632/3zggb53m4x.3 [7])
Data file 65	Castbox	Archive file format (.rar) containing PCAP files	Mendeley Data (https://doi.org/10.17632/3zggb53m4x.3 [7])
Data file 66	Clash of Clans	Archive file format (.rar) containing PCAP files	Mendeley Data (https://doi.org/10.17632/3zggb53m4x.3 [7])
Data file 67	Clubhouse	Archive file format (.rar) containing PCAP files	Mendeley Data (https://doi.org/10.17632/3zggb53m4x.3 [7])
Data file 68	Coursera	Archive file format (.rar) containing PCAP files	Mendeley Data (https://doi.org/10.17632/3zggb53m4x.3 [7])
Data file 69	Digikala	Archive file format (.rar) containing PCAP files	Mendeley Data (https://doi.org/10.17632/3zggb53m4x.3 [7])
Data file 70	Discord	Archive file format (.rar) containing PCAP files	Mendeley Data (https://doi.org/10.17632/3zggb53m4x.3 [7])
Data file 71	Divar	Archive file format (.rar) containing PCAP files	Mendeley Data (https://doi.org/10.17632/3zggb53m4x.3 [7])
Data file 72	Dropbox	Archive file format (.rar) containing PCAP files	Mendeley Data (https://doi.org/10.17632/3zggb53m4x.3 [7])
Data file 73	Duolingo	Archive file format (.rar) containing PCAP files	Mendeley Data (https://doi.org/10.17632/3zggb53m4x.3 [7])
Data file 74	Eitaa	Archive file format (.rar) containing PCAP files	Mendeley Data (https://doi.org/10.17632/3zggb53m4x.3 [7])
Data file 75	Ewano	Archive file format (.rar) containing PCAP files	Mendeley Data (https://doi.org/10.17632/3zggb53m4x.3 [7])
Data file 76	Facebook Lite	Archive file format (.rar) containing PCAP files	Mendeley Data (https://doi.org/10.17632/3zggb53m4x.3 [7])
Data file 77	Facelab	Archive file format (.rar) containing PCAP files	Mendeley Data (https://doi.org/10.17632/3zggb53m4x.3 [7])
Data file 78	Fidibo	Archive file format (.rar) containing PCAP files	Mendeley Data (https://doi.org/10.17632/3zggb53m4x.3 [7])
Data file 79	Filimo	Archive file format (.rar) containing PCAP files	Mendeley Data (https://doi.org/10.17632/3zggb53m4x.3 [7])
Data file 80	Firefox Browser	Archive file format (.rar) containing PCAP files	Mendeley Data (https://doi.org/10.17632/3zggb53m4x.3 [7])
Data file 81	Football Strike	Archive file format (.rar) containing PCAP files	Mendeley Data (https://doi.org/10.17632/3zggb53m4x.3 [7])
Data file 82	Gmail	Archive file format (.rar) containing PCAP files	Mendeley Data (https://doi.org/10.17632/3zggb53m4x.3 [7])
Data file 83	Goodreads	Archive file format (.rar) containing PCAP files	Mendeley Data (https://doi.org/10.17632/3zggb53m4x.3 [7])
Data file 84	Google Chrome	Archive file format (.rar) containing PCAP files	Mendeley Data (https://doi.org/10.17632/3zggb53m4x.3 [7])
Data file 85	Google Maps	Archive file format (.rar) containing PCAP files	Mendeley Data (https://doi.org/10.17632/3zggb53m4x.3 [7])
Data file 86	Google Meet	Archive file format (.rar) containing PCAP files	Mendeley Data (https://doi.org/10.17632/3zggb53m4x.3 [7])
Data file 87	Google Play Store	Archive file format (.rar) containing PCAP files	Mendeley Data (https://doi.org/10.17632/3zggb53m4x.3 [7])
Data file 88	iGap	Archive file format (.rar) containing PCAP files	Mendeley Data (https://doi.org/10.17632/3zggb53m4x.3 [7])
Data file 89	InSave	Archive file format (.rar) containing PCAP files	Mendeley Data (https://doi.org/10.17632/3zggb53m4x.3 [7])
Data file 90	Instagram	Archive file format (.rar) containing PCAP files	Mendeley Data (https://doi.org/10.17632/3zggb53m4x.3 [7])
Data file 91	Likee	Archive file format (.rar) containing PCAP files	Mendeley Data (https://doi.org/10.17632/3zggb53m4x.3 [7])
Data file 92	LinkedIn	Archive file format (.rar) containing PCAP files	Mendeley Data (https://doi.org/10.17632/3zggb53m4x.3 [7])
Data file 93	Mafioso	Archive file format (.rar) containing PCAP files	Mendeley Data (https://doi.org/10.17632/3zggb53m4x.3 [7])
Data file 94	Memrise	Archive file format (.rar) containing PCAP files	Mendeley Data (https://doi.org/10.17632/3zggb53m4x.3 [7])
Data file 95	Mencherz	Archive file format (.rar) containing PCAP files	Mendeley Data (https://doi.org/10.17632/3zggb53m4x.3 [7])
Data file 96	Microsoft Outlook	Archive file format (.rar) containing PCAP files	Mendeley Data (https://doi.org/10.17632/3zggb53m4x.3 [7])
Data file 97	Myket	Archive file format (.rar) containing PCAP files	Mendeley Data (https://doi.org/10.17632/3zggb53m4x.3 [7])
Data file 98	Neshan	Archive file format (.rar) containing PCAP files	Mendeley Data (https://doi.org/10.17632/3zggb53m4x.3 [7])
Data file 99	OneDrive	Archive file format (.rar) containing PCAP files	Mendeley Data (https://doi.org/10.17632/3zggb53m4x.3 [7])
Data file 100	Pinterest	Archive file format (.rar) containing PCAP files	Mendeley Data (https://doi.org/10.17632/3zggb53m4x.3 [7])
Data file 101	Quiz Of Kings	Archive file format (.rar) containing PCAP files	Mendeley Data (https://doi.org/10.17632/3zggb53m4x.3 [7])
Data file 102	Radio Javan	Archive file format (.rar) containing PCAP files	Mendeley Data (https://doi.org/10.17632/3zggb53m4x.3 [7])
Data file 103	Shaypur	Archive file format (.rar) containing PCAP files	Mendeley Data (https://doi.org/10.17632/3zggb53m4x.3 [7])
Data file 104	Skype	Archive file format (.rar) containing PCAP files	Mendeley Data (https://doi.org/10.17632/3zggb53m4x.3 [7])
Data file 105	Snapchat	Archive file format (.rar) containing PCAP files	Mendeley Data (https://doi.org/10.17632/3zggb53m4x.3 [7])
Data file 106	Snapp	Archive file format (.rar) containing PCAP files	Mendeley Data (https://doi.org/10.17632/3zggb53m4x.3 [7])
Data file 107	Soroush Plus Messenger	Archive file format (.rar) containing PCAP files	Mendeley Data (https://doi.org/10.17632/3zggb53m4x.3 [7])
Data file 108	Spotify	Archive file format (.rar) containing PCAP files	Mendeley Data (https://doi.org/10.17632/3zggb53m4x.3 [7])
Data file 109	Taghche	Archive file format (.rar) containing PCAP files	Mendeley Data (https://doi.org/10.17632/3zggb53m4x.3 [7])
Data file 110	Tapsi	Archive file format (.rar) containing PCAP files	Mendeley Data (https://doi.org/10.17632/3zggb53m4x.3 [7])
Data file 111	Telegram	Archive file format (.rar) containing PCAP files	Mendeley Data (https://doi.org/10.17632/3zggb53m4x.3 [7])
Data file 112	Telewebion	Archive file format (.rar) containing PCAP files	Mendeley Data (https://doi.org/10.17632/3zggb53m4x.3 [7])
Data file 113	ToonMe	Archive file format (.rar) containing PCAP files	Mendeley Data (https://doi.org/10.17632/3zggb53m4x.3 [7])
Data file 114	Torob	Archive file format (.rar) containing PCAP files	Mendeley Data (https://doi.org/10.17632/3zggb53m4x.3 [7])
Data file 115	Twitter	Archive file format (.rar) containing PCAP files	Mendeley Data (https://doi.org/10.17632/3zggb53m4x.3 [7])
Data file 116	Waze	Archive file format (.rar) containing PCAP files	Mendeley Data (https://doi.org/10.17632/3zggb53m4x.3 [7])
Data file 117	Whatsapp Business	Archive file format (.rar) containing PCAP files	Mendeley Data (https://doi.org/10.17632/3zggb53m4x.3 [7])
Data file 118	Whatsapp Messenger	Archive file format (.rar) containing PCAP files	Mendeley Data (https://doi.org/10.17632/3zggb53m4x.3 [7])
Data file 119	Youtube	Archive file format (.rar) containing PCAP files	Mendeley Data (https://doi.org/10.17632/3zggb53m4x.3 [7])
Data file 120	AP—Asan Pardakht	Archive file format (.rar) containing PCAP files	Mendeley Data (https://doi.org/10.17632/gp8r347j38.3 [8])
Data file 121	Aparat	Archive file format (.rar) containing PCAP files	Mendeley Data (https://doi.org/10.17632/gp8r347j38.3 [8])
Data file 122	Balad	Archive file format (.rar) containing PCAP files	Mendeley Data (https://doi.org/10.17632/gp8r347j38.3 [8])
Data file 123	Bazaar	Archive file format (.rar) containing PCAP files	Mendeley Data (https://doi.org/10.17632/gp8r347j38.3 [8])
Data file 124	Castbox	Archive file format (.rar) containing PCAP files	Mendeley Data (https://doi.org/10.17632/gp8r347j38.3 [8])
Data file 125	Clash of Clans	Archive file format (.rar) containing PCAP files	Mendeley Data (https://doi.org/10.17632/gp8r347j38.3 [8])
Data file 126	Clubhouse	Archive file format (.rar) containing PCAP files	Mendeley Data (https://doi.org/10.17632/gp8r347j38.3 [8])
Data file 127	Coursera	Archive file format (.rar) containing PCAP files	Mendeley Data (https://doi.org/10.17632/gp8r347j38.3 [8])
Data file 128	Digikala	Archive file format (.rar) containing PCAP files	Mendeley Data (https://doi.org/10.17632/gp8r347j38.3 [8])
Data file 129	Discord	Archive file format (.rar) containing PCAP files	Mendeley Data (https://doi.org/10.17632/gp8r347j38.3 [8])
Data file 130	Divar	Archive file format (.rar) containing PCAP files	Mendeley Data (https://doi.org/10.17632/gp8r347j38.3 [8])
Data file 131	Dropbox	Archive file format (.rar) containing PCAP files	Mendeley Data (https://doi.org/10.17632/gp8r347j38.3 [8])
Data file 132	Duolingo	Archive file format (.rar) containing PCAP files	Mendeley Data (https://doi.org/10.17632/gp8r347j38.3 [8])
Data file 133	Eitaa	Archive file format (.rar) containing PCAP files	Mendeley Data (https://doi.org/10.17632/gp8r347j38.3 [8])
Data file 134	Ewano	Archive file format (.rar) containing PCAP files	Mendeley Data (https://doi.org/10.17632/gp8r347j38.3 [8])
Data file 135	Facebook Lite	Archive file format (.rar) containing PCAP files	Mendeley Data (https://doi.org/10.17632/gp8r347j38.3 [8])
Data file 136	Facelab	Archive file format (.rar) containing PCAP files	Mendeley Data (https://doi.org/10.17632/gp8r347j38.3 [8])
Data file 137	Fidibo	Archive file format (.rar) containing PCAP files	Mendeley Data (https://doi.org/10.17632/gp8r347j38.3 [8])
Data file 138	Filimo	Archive file format (.rar) containing PCAP files	Mendeley Data (https://doi.org/10.17632/gp8r347j38.3 [8])
Data file 139	Firefox Browser	Archive file format (.rar) containing PCAP files	Mendeley Data (https://doi.org/10.17632/gp8r347j38.3 [8])
Data file 140	Football Strike	Archive file format (.rar) containing PCAP files	Mendeley Data (https://doi.org/10.17632/gp8r347j38.3 [8])
Data file 141	Gmail	Archive file format (.rar) containing PCAP files	Mendeley Data (https://doi.org/10.17632/gp8r347j38.3 [8])
Data file 142	Goodreads	Archive file format (.rar) containing PCAP files	Mendeley Data (https://doi.org/10.17632/gp8r347j38.3 [8])
Data file 143	Google Chrome	Archive file format (.rar) containing PCAP files	Mendeley Data (https://doi.org/10.17632/gp8r347j38.3 [8])
Data file 144	Google Maps	Archive file format (.rar) containing PCAP files	Mendeley Data (https://doi.org/10.17632/gp8r347j38.3 [8])
Data file 145	Google Meet	Archive file format (.rar) containing PCAP files	Mendeley Data (https://doi.org/10.17632/gp8r347j38.3 [8])
Data file 146	Google Play Store	Archive file format (.rar) containing PCAP files	Mendeley Data (https://doi.org/10.17632/gp8r347j38.3 [8])
Data file 147	iGap	Archive file format (.rar) containing PCAP files	Mendeley Data (https://doi.org/10.17632/gp8r347j38.3 [8])
Data file 148	InSave	Archive file format (.rar) containing PCAP files	Mendeley Data (https://doi.org/10.17632/gp8r347j38.3 [8])
Data file 149	Instagram	Archive file format (.rar) containing PCAP files	Mendeley Data (https://doi.org/10.17632/gp8r347j38.3 [8])
Data file 150	Likee	Archive file format (.rar) containing PCAP files	Mendeley Data (https://doi.org/10.17632/gp8r347j38.3 [8])
Data file 151	LinkedIn	Archive file format (.rar) containing PCAP files	Mendeley Data (https://doi.org/10.17632/gp8r347j38.3 [8])
Data file 152	Mafioso	Archive file format (.rar) containing PCAP files	Mendeley Data (https://doi.org/10.17632/gp8r347j38.3 [8])
Data file 153	Memrise	Archive file format (.rar) containing PCAP files	Mendeley Data (https://doi.org/10.17632/gp8r347j38.3 [8])
Data file 154	Mencherz	Archive file format (.rar) containing PCAP files	Mendeley Data (https://doi.org/10.17632/gp8r347j38.3 [8])
Data file 155	Microsoft Outlook	Archive file format (.rar) containing PCAP files	Mendeley Data (https://doi.org/10.17632/gp8r347j38.3 [8])
Data file 156	Myket	Archive file format (.rar) containing PCAP files	Mendeley Data (https://doi.org/10.17632/gp8r347j38.3 [8])
Data file 157	Neshan	Archive file format (.rar) containing PCAP files	Mendeley Data (https://doi.org/10.17632/gp8r347j38.3 [8])
Data file 158	OneDrive	Archive file format (.rar) containing PCAP files	Mendeley Data (https://doi.org/10.17632/gp8r347j38.3 [8])
Data file 159	Pinterest	Archive file format (.rar) containing PCAP files	Mendeley Data (https://doi.org/10.17632/gp8r347j38.3 [8])
Data file 160	Quiz Of Kings	Archive file format (.rar) containing PCAP files	Mendeley Data (https://doi.org/10.17632/gp8r347j38.3 [8])
Data file 161	Radio Javan	Archive file format (.rar) containing PCAP files	Mendeley Data (https://doi.org/10.17632/gp8r347j38.3 [8])
Data file 162	Shaypur	Archive file format (.rar) containing PCAP files	Mendeley Data (https://doi.org/10.17632/gp8r347j38.3 [8])
Data file 163	Skype	Archive file format (.rar) containing PCAP files	Mendeley Data (https://doi.org/10.17632/gp8r347j38.3 [8])
Data file 164	Snapchat	Archive file format (.rar) containing PCAP files	Mendeley Data (https://doi.org/10.17632/gp8r347j38.3 [8])
Data file 165	Snapp	Archive file format (.rar) containing PCAP files	Mendeley Data (https://doi.org/10.17632/gp8r347j38.3 [8])
Data file 166	Soroush Plus Messenger	Archive file format (.rar) containing PCAP files	Mendeley Data (https://doi.org/10.17632/gp8r347j38.3 [8])
Data file 167	Spotify	Archive file format (.rar) containing PCAP files	Mendeley Data (https://doi.org/10.17632/gp8r347j38.3 [8])
Data file 168	Taghche	Archive file format (.rar) containing PCAP files	Mendeley Data (https://doi.org/10.17632/gp8r347j38.3 [8])
Data file 169	Tapsi	Archive file format (.rar) containing PCAP files	Mendeley Data (https://doi.org/10.17632/gp8r347j38.3 [8])
Data file 170	Telegram	Archive file format (.rar) containing PCAP files	Mendeley Data (https://doi.org/10.17632/gp8r347j38.3 [8])
Data file 171	Telewebion	Archive file format (.rar) containing PCAP files	Mendeley Data (https://doi.org/10.17632/gp8r347j38.3 [8])
Data file 172	ToonMe	Archive file format (.rar) containing PCAP files	Mendeley Data (https://doi.org/10.17632/gp8r347j38.3 [8])
Data file 173	Torob	Archive file format (.rar) containing PCAP files	Mendeley Data (https://doi.org/10.17632/gp8r347j38.3 [8])
Data file 174	Twitter	Archive file format (.rar) containing PCAP files	Mendeley Data (https://doi.org/10.17632/gp8r347j38.3 [8])
Data file 175	Waze	Archive file format (.rar) containing PCAP files	Mendeley Data (https://doi.org/10.17632/gp8r347j38.3 [8])
Data file 176	Whatsapp Business	Archive file format (.rar) containing PCAP files	Mendeley Data (https://doi.org/10.17632/gp8r347j38.3 [8])
Data file 177	Whatsapp Messenger	Archive file format (.rar) containing PCAP files	Mendeley Data (https://doi.org/10.17632/gp8r347j38.3 [8])
Data file 178	Youtube	Archive file format (.rar) containing PCAP files	Mendeley Data (https://doi.org/10.17632/gp8r347j38.3 [8])
Data file 179	Adobe Connect	Archive file format (.rar) containing PCAP files	Mendeley Data (https://doi.org/10.17632/mcmf627yh5.3 [9])
Data file 180	AP—Asan Pardakht	Archive file format (.rar) containing PCAP files	Mendeley Data (https://doi.org/10.17632/mcmf627yh5.3 [9])
Data file 181	Aparat	Archive file format (.rar) containing PCAP files	Mendeley Data (https://doi.org/10.17632/mcmf627yh5.3 [9])
Data file 182	Balad	Archive file format (.rar) containing PCAP files	Mendeley Data (https://doi.org/10.17632/mcmf627yh5.3 [9])
Data file 183	Bazaar	Archive file format (.rar) containing PCAP files	Mendeley Data (https://doi.org/10.17632/mcmf627yh5.3 [9])
Data file 184	Castbox	Archive file format (.rar) containing PCAP files	Mendeley Data (https://doi.org/10.17632/mcmf627yh5.3 [9])
Data file 185	Clash of Clans	Archive file format (.rar) containing PCAP files	Mendeley Data (https://doi.org/10.17632/mcmf627yh5.3 [9])
Data file 186	Clubhouse	Archive file format (.rar) containing PCAP files	Mendeley Data (https://doi.org/10.17632/mcmf627yh5.3 [9])
Data file 187	Coursera	Archive file format (.rar) containing PCAP files	Mendeley Data (https://doi.org/10.17632/mcmf627yh5.3 [9])
Data file 188	Digikala	Archive file format (.rar) containing PCAP files	Mendeley Data (https://doi.org/10.17632/mcmf627yh5.3 [9])
Data file 189	Discord	Archive file format (.rar) containing PCAP files	Mendeley Data (https://doi.org/10.17632/mcmf627yh5.3 [9])
Data file 190	Divar	Archive file format (.rar) containing PCAP files	Mendeley Data (https://doi.org/10.17632/mcmf627yh5.3 [9])
Data file 191	Dropbox	Archive file format (.rar) containing PCAP files	Mendeley Data (https://doi.org/10.17632/mcmf627yh5.3 [9])
Data file 192	Duolingo	Archive file format (.rar) containing PCAP files	Mendeley Data (https://doi.org/10.17632/mcmf627yh5.3 [9])
Data file 193	Eitaa	Archive file format (.rar) containing PCAP files	Mendeley Data (https://doi.org/10.17632/mcmf627yh5.3 [9])
Data file 194	Ewano	Archive file format (.rar) containing PCAP files	Mendeley Data (https://doi.org/10.17632/mcmf627yh5.3 [9])
Data file 195	Facebook Lite	Archive file format (.rar) containing PCAP files	Mendeley Data (https://doi.org/10.17632/mcmf627yh5.3 [9])
Data file 196	Facelab	Archive file format (.rar) containing PCAP files	Mendeley Data (https://doi.org/10.17632/mcmf627yh5.3 [9])
Data file 197	Fidibo	Archive file format (.rar) containing PCAP files	Mendeley Data (https://doi.org/10.17632/mcmf627yh5.3 [9])
Data file 198	Filimo	Archive file format (.rar) containing PCAP files	Mendeley Data (https://doi.org/10.17632/mcmf627yh5.3 [9])
Data file 199	Firefox Browser	Archive file format (.rar) containing PCAP files	Mendeley Data (https://doi.org/10.17632/mcmf627yh5.3 [9])
Data file 200	Football Strike	Archive file format (.rar) containing PCAP files	Mendeley Data (https://doi.org/10.17632/mcmf627yh5.3 [9])
Data file 201	Gmail	Archive file format (.rar) containing PCAP files	Mendeley Data (https://doi.org/10.17632/mcmf627yh5.3 [9])
Data file 202	Goodreads	Archive file format (.rar) containing PCAP files	Mendeley Data (https://doi.org/10.17632/mcmf627yh5.3 [9])
Data file 203	Google Chrome	Archive file format (.rar) containing PCAP files	Mendeley Data (https://doi.org/10.17632/mcmf627yh5.3 [9])
Data file 204	Google Maps	Archive file format (.rar) containing PCAP files	Mendeley Data (https://doi.org/10.17632/mcmf627yh5.3 [9])
Data file 205	Google Meet	Archive file format (.rar) containing PCAP files	Mendeley Data (https://doi.org/10.17632/mcmf627yh5.3 [9])
Data file 206	Google Play Store	Archive file format (.rar) containing PCAP files	Mendeley Data (https://doi.org/10.17632/mcmf627yh5.3 [9])
Data file 207	iGap	Archive file format (.rar) containing PCAP files	Mendeley Data (https://doi.org/10.17632/mcmf627yh5.3 [9])
Data file 208	InSave	Archive file format (.rar) containing PCAP files	Mendeley Data (https://doi.org/10.17632/mcmf627yh5.3 [9])
Data file 209	Instagram	Archive file format (.rar) containing PCAP files	Mendeley Data (https://doi.org/10.17632/mcmf627yh5.3 [9])
Data file 210	Likee	Archive file format (.rar) containing PCAP files	Mendeley Data (https://doi.org/10.17632/mcmf627yh5.3 [9])
Data file 211	LinkedIn	Archive file format (.rar) containing PCAP files	Mendeley Data (https://doi.org/10.17632/mcmf627yh5.3 [9])
Data file 212	Mafioso	Archive file format (.rar) containing PCAP files	Mendeley Data (https://doi.org/10.17632/mcmf627yh5.3 [9])
Data file 213	Memrise	Archive file format (.rar) containing PCAP files	Mendeley Data (https://doi.org/10.17632/mcmf627yh5.3 [9])
Data file 214	Mencherz	Archive file format (.rar) containing PCAP files	Mendeley Data (https://doi.org/10.17632/mcmf627yh5.3 [9])
Data file 215	Microsoft Outlook	Archive file format (.rar) containing PCAP files	Mendeley Data (https://doi.org/10.17632/mcmf627yh5.3 [9])
Data file 216	Myket	Archive file format (.rar) containing PCAP files	Mendeley Data (https://doi.org/10.17632/mcmf627yh5.3 [9])
Data file 217	Neshan	Archive file format (.rar) containing PCAP files	Mendeley Data (https://doi.org/10.17632/mcmf627yh5.3 [9])
Data file 218	OneDrive	Archive file format (.rar) containing PCAP files	Mendeley Data (https://doi.org/10.17632/mcmf627yh5.3 [9])
Data file 219	Pinterest	Archive file format (.rar) containing PCAP files	Mendeley Data (https://doi.org/10.17632/mcmf627yh5.3 [9])
Data file 220	Quiz Of Kings	Archive file format (.rar) containing PCAP files	Mendeley Data (https://doi.org/10.17632/mcmf627yh5.3 [9])
Data file 221	Radio Javan	Archive file format (.rar) containing PCAP files	Mendeley Data (https://doi.org/10.17632/mcmf627yh5.3 [9])
Data file 222	Shaypur	Archive file format (.rar) containing PCAP files	Mendeley Data (https://doi.org/10.17632/mcmf627yh5.3 [9])
Data file 223	Skype	Archive file format (.rar) containing PCAP files	Mendeley Data (https://doi.org/10.17632/mcmf627yh5.3 [9])
Data file 224	Snapchat	Archive file format (.rar) containing PCAP files	Mendeley Data (https://doi.org/10.17632/mcmf627yh5.3 [9])
Data file 225	Snapp	Archive file format (.rar) containing PCAP files	Mendeley Data (https://doi.org/10.17632/mcmf627yh5.3 [9])
Data file 226	Soroush Plus Messenger	Archive file format (.rar) containing PCAP files	Mendeley Data (https://doi.org/10.17632/mcmf627yh5.3 [9])
Data file 227	Spotify	Archive file format (.rar) containing PCAP files	Mendeley Data (https://doi.org/10.17632/mcmf627yh5.3 [9])
Data file 228	Taghche	Archive file format (.rar) containing PCAP files	Mendeley Data (https://doi.org/10.17632/mcmf627yh5.3 [9])
Data file 229	Tapsi	Archive file format (.rar) containing PCAP files	Mendeley Data (https://doi.org/10.17632/mcmf627yh5.3 [9])
Data file 230	Telegram	Archive file format (.rar) containing PCAP files	Mendeley Data (https://doi.org/10.17632/mcmf627yh5.3 [9])
Data file 231	Telewebion	Archive file format (.rar) containing PCAP files	Mendeley Data (https://doi.org/10.17632/mcmf627yh5.3 [9])
Data file 232	ToonMe	Archive file format (.rar) containing PCAP files	Mendeley Data (https://doi.org/10.17632/mcmf627yh5.3 [9])
Data file 233	Torob	Archive file format (.rar) containing PCAP files	Mendeley Data (https://doi.org/10.17632/mcmf627yh5.3 [9])
Data file 234	Twitter	Archive file format (.rar) containing PCAP files	Mendeley Data (https://doi.org/10.17632/mcmf627yh5.3 [9])
Data file 235	Waze	Archive file format (.rar) containing PCAP files	Mendeley Data (https://doi.org/10.17632/mcmf627yh5.3 [9])
Data file 236	Whatsapp Messenger	Archive file format (.rar) containing PCAP files	Mendeley Data (https://doi.org/10.17632/mcmf627yh5.3 [9])
Data file 237	Youtube	Archive file format (.rar) containing PCAP files	Mendeley Data (https://doi.org/10.17632/mcmf627yh5.3 [9])
Data file 238	Adobe Connect	Archive file format (.rar) containing PCAP files	Mendeley Data (https://doi.org/10.17632/gdtnnfyr7s.3 [10])
Data file 239	AP—Asan Pardakht	Archive file format (.rar) containing PCAP files	Mendeley Data (https://doi.org/10.17632/gdtnnfyr7s.3 [10])
Data file 240	Aparat	Archive file format (.rar) containing PCAP files	Mendeley Data (https://doi.org/10.17632/gdtnnfyr7s.3 [10])
Data file 241	Balad	Archive file format (.rar) containing PCAP files	Mendeley Data (https://doi.org/10.17632/gdtnnfyr7s.3 [10])
Data file 242	Bazaar	Archive file format (.rar) containing PCAP files	Mendeley Data (https://doi.org/10.17632/gdtnnfyr7s.3 [10])
Data file 243	Castbox	Archive file format (.rar) containing PCAP files	Mendeley Data (https://doi.org/10.17632/gdtnnfyr7s.3 [10])
Data file 244	Clash of Clans	Archive file format (.rar) containing PCAP files	Mendeley Data (https://doi.org/10.17632/gdtnnfyr7s.3 [10])
Data file 245	Clubhouse	Archive file format (.rar) containing PCAP files	Mendeley Data (https://doi.org/10.17632/gdtnnfyr7s.3 [10])
Data file 246	Coursera	Archive file format (.rar) containing PCAP files	Mendeley Data (https://doi.org/10.17632/gdtnnfyr7s.3 [10])
Data file 247	Digikala	Archive file format (.rar) containing PCAP files	Mendeley Data (https://doi.org/10.17632/gdtnnfyr7s.3 [10])
Data file 248	Divar	Archive file format (.rar) containing PCAP files	Mendeley Data (https://doi.org/10.17632/gdtnnfyr7s.3 [10])
Data file 249	Duolingo	Archive file format (.rar) containing PCAP files	Mendeley Data (https://doi.org/10.17632/gdtnnfyr7s.3 [10])
Data file 250	Eitaa	Archive file format (.rar) containing PCAP files	Mendeley Data (https://doi.org/10.17632/gdtnnfyr7s.3 [10])
Data file 251	Ewano	Archive file format (.rar) containing PCAP files	Mendeley Data (https://doi.org/10.17632/gdtnnfyr7s.3 [10])
Data file 252	Facebook Lite	Archive file format (.rar) containing PCAP files	Mendeley Data (https://doi.org/10.17632/gdtnnfyr7s.3 [10])
Data file 253	Facelab	Archive file format (.rar) containing PCAP files	Mendeley Data (https://doi.org/10.17632/gdtnnfyr7s.3 [10])
Data file 254	Fidibo	Archive file format (.rar) containing PCAP files	Mendeley Data (https://doi.org/10.17632/gdtnnfyr7s.3 [10])
Data file 255	Filimo	Archive file format (.rar) containing PCAP files	Mendeley Data (https://doi.org/10.17632/gdtnnfyr7s.3 [10])
Data file 256	Firefox Browser	Archive file format (.rar) containing PCAP files	Mendeley Data (https://doi.org/10.17632/gdtnnfyr7s.3 [10])
Data file 257	Football Strike	Archive file format (.rar) containing PCAP files	Mendeley Data (https://doi.org/10.17632/gdtnnfyr7s.3 [10])
Data file 258	Gmail	Archive file format (.rar) containing PCAP files	Mendeley Data (https://doi.org/10.17632/gdtnnfyr7s.3 [10])
Data file 259	Goodreads	Archive file format (.rar) containing PCAP files	Mendeley Data (https://doi.org/10.17632/gdtnnfyr7s.3 [10])
Data file 260	Google Chrome	Archive file format (.rar) containing PCAP files	Mendeley Data (https://doi.org/10.17632/gdtnnfyr7s.3 [10])
Data file 261	Google Maps	Archive file format (.rar) containing PCAP files	Mendeley Data (https://doi.org/10.17632/gdtnnfyr7s.3 [10])
Data file 262	Google Meet	Archive file format (.rar) containing PCAP files	Mendeley Data (https://doi.org/10.17632/gdtnnfyr7s.3 [10])
Data file 263	Google Play Store	Archive file format (.rar) containing PCAP files	Mendeley Data (https://doi.org/10.17632/gdtnnfyr7s.3 [10])
Data file 264	iGap	Archive file format (.rar) containing PCAP files	Mendeley Data (https://doi.org/10.17632/gdtnnfyr7s.3 [10])
Data file 265	InSave	Archive file format (.rar) containing PCAP files	Mendeley Data (https://doi.org/10.17632/gdtnnfyr7s.3 [10])
Data file 266	Instagram	Archive file format (.rar) containing PCAP files	Mendeley Data (https://doi.org/10.17632/gdtnnfyr7s.3 [10])
Data file 267	Likee	Archive file format (.rar) containing PCAP files	Mendeley Data (https://doi.org/10.17632/gdtnnfyr7s.3 [10])
Data file 268	Mafioso	Archive file format (.rar) containing PCAP files	Mendeley Data (https://doi.org/10.17632/gdtnnfyr7s.3 [10])
Data file 269	Memrise	Archive file format (.rar) containing PCAP files	Mendeley Data (https://doi.org/10.17632/gdtnnfyr7s.3 [10])
Data file 270	Mencherz	Archive file format (.rar) containing PCAP files	Mendeley Data (https://doi.org/10.17632/gdtnnfyr7s.3 [10])
Data file 271	Myket	Archive file format (.rar) containing PCAP files	Mendeley Data (https://doi.org/10.17632/gdtnnfyr7s.3 [10])
Data file 272	Neshan	Archive file format (.rar) containing PCAP files	Mendeley Data (https://doi.org/10.17632/gdtnnfyr7s.3 [10])
Data file 273	OneDrive	Archive file format (.rar) containing PCAP files	Mendeley Data (https://doi.org/10.17632/gdtnnfyr7s.3 [10])
Data file 274	Pinterest	Archive file format (.rar) containing PCAP files	Mendeley Data (https://doi.org/10.17632/gdtnnfyr7s.3 [10])
Data file 275	Quiz Of Kings	Archive file format (.rar) containing PCAP files	Mendeley Data (https://doi.org/10.17632/gdtnnfyr7s.3 [10])
Data file 276	Radio Javan	Archive file format (.rar) containing PCAP files	Mendeley Data (https://doi.org/10.17632/gdtnnfyr7s.3 [10])
Data file 277	Shaypur	Archive file format (.rar) containing PCAP files	Mendeley Data (https://doi.org/10.17632/gdtnnfyr7s.3 [10])
Data file 278	Skype	Archive file format (.rar) containing PCAP files	Mendeley Data (https://doi.org/10.17632/gdtnnfyr7s.3 [10])
Data file 279	Snapchat	Archive file format (.rar) containing PCAP files	Mendeley Data (https://doi.org/10.17632/gdtnnfyr7s.3 [10])
Data file 280	Snapp	Archive file format (.rar) containing PCAP files	Mendeley Data (https://doi.org/10.17632/gdtnnfyr7s.3 [10])
Data file 281	Soroush Plus Messenger	Archive file format (.rar) containing PCAP files	Mendeley Data (https://doi.org/10.17632/gdtnnfyr7s.3 [10])
Data file 282	Spotify	Archive file format (.rar) containing PCAP files	Mendeley Data (https://doi.org/10.17632/gdtnnfyr7s.3 [10])
Data file 283	Taghche	Archive file format (.rar) containing PCAP files	Mendeley Data (https://doi.org/10.17632/gdtnnfyr7s.3 [10])
Data file 284	Tapsi	Archive file format (.rar) containing PCAP files	Mendeley Data (https://doi.org/10.17632/gdtnnfyr7s.3 [10])
Data file 285	Telegram	Archive file format (.rar) containing PCAP files	Mendeley Data (https://doi.org/10.17632/gdtnnfyr7s.3 [10])
Data file 286	ToonMe	Archive file format (.rar) containing PCAP files	Mendeley Data (https://doi.org/10.17632/gdtnnfyr7s.3 [10])
Data file 287	Torob	Archive file format (.rar) containing PCAP files	Mendeley Data (https://doi.org/10.17632/gdtnnfyr7s.3 [10])
Data file 288	Whatsapp Messenger	Archive file format (.rar) containing PCAP files	Mendeley Data (https://doi.org/10.17632/gdtnnfyr7s.3 [10])
Data file 289	Youtube	Archive file format (.rar) containing PCAP files	Mendeley Data (https://doi.org/10.17632/gdtnnfyr7s.3 [10])
Data file 290	DetailedDescription	Portable document format (.pdf)	Mendeley Data (https://doi.org/10.17632/4sgt9tjs4w.7 [11])
Data file 291	filter_background_traffic	Python script file (.py)	Mendeley Data (https://doi.org/10.17632/4sgt9tjs4w.7 [11])
Data file 292	extract_PCAP_file_properties	Python script file (.py)	Mendeley Data (https://doi.org/10.17632/4sgt9tjs4w.7 [11])

1 and references [6–11] for details and links to the data. Overview of data files/data sets

## Abbreviations

PCAP: Packet capture
PC: Personal computer
VPN: Virtual private network
