# Supplementary material for: ITC-Net-blend-60: a comprehensive dataset for robust network traffic classification in diverse environments
Source: BMC Res Notes. 2024 Jun 15;17:165. doi: 10.1186/s13104-024-06817-5 (PMC11179189; doi:10.1186/s13104-024-06817-5)
Supplement: Supplementary file 1 — Additional file1 (DOCX 370 KB) [file 13104_2024_6817_MOESM1_ESM.docx]

**ITC-Net-Blend-60: A Comprehensive Dataset for Robust Network Traffic Classification in Diverse Environments.**

# **Experimental Design, Materials, and Methods**

The methodology employed for collecting the dataset comprised three main stages: Application Selection, Traffic Capture Setup, and Traffic Generation. In the application selection phase, we chose the applications to monitor. Next, in the traffic capture setup phase, we set up the framework to capture network traffic data. Finally, in the traffic generation phase, we generated the actual network traffic data from the selected applications. Details on each phase will be provided in subsequent sections.

## Application Selection

Since it is not feasible to capture the traffic of all applications, we considered the top 300 free Android apps listed in October 2021 in the Google Play Store and two major Iranian Android app markets, Cafe Bazaar^[[1]](#footnote-1)^, and Myket^[[2]](#footnote-2)^. From these, we selected a subset of 60 applications based on two criteria:

- The application's main activity relied on an Internet connection
- The application generated traffic through user interactions

The chosen applications spanned 16 different categories. The complete list of the selected 60 applications and additional information can be found in **S Table A1** in Appendix.

## Traffic Capture Setup

As depicted in **S Figure 1**, our traffic capture setup included a smartphone and a laptop. We used a laptop running Windows 10 with an internal dual-band network card and installed Wireshark software on it. The laptop was connected to the internet and shared its connection with the smartphone via a hotspot. Then we configured Wireshark to capture traffic through the "Local Area Connection" interface. This setup enabled the smartphone to access the internet through the laptop's connection, allowing Wireshark to capture the smartphone's network traffic.


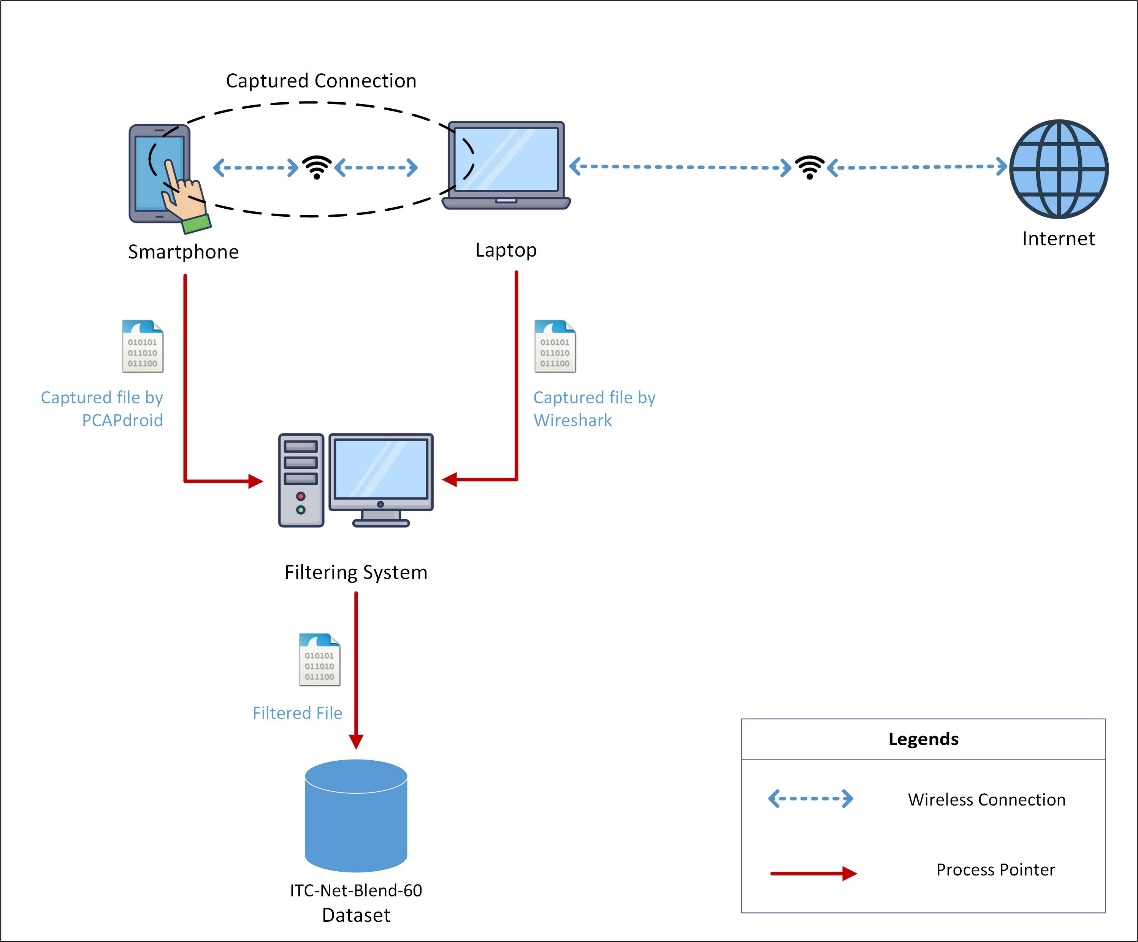


S Figure 1: Traffic capture setup.

The traffic captured by Wireshark contained significant background traffic. To isolate the target application's network traffic, we installed PCAPdroid^[[3]](#footnote-3)^ on the smartphone. Since root access could modify application behavior, we used PCAPdroid in non-root mode. In this mode, PCAPdroid does not use a remote VPN server; instead, it simulates a VPN to capture the network traffic and processes data locally on the device.

While PCAPdroid can capture an individual application's traffic, it modifies network layers 3 and 4 of packets, preventing its independent use. Therefore, we used Wireshark and PCAPdroid simultaneously to record the target application's traffic. Wireshark provided an unaltered packet capture, while PCAPdroid isolated traffic originating from the application. The two tools complemented each other to provide an accurate capture of the traffic.

Unfortunately, several applications had restrictions on accessing their servers in Iran, which required us to use a VPN connection to execute them properly. These applications are identified in Table A. For this purpose, we installed the free version of ProtonVPN^[[4]](#footnote-4)^ on the laptop and configured its protocol to OpenVPN-UDP while setting the VPN network driver to the TAP adapter. To ensure that recorded packets were not modified by the VPN, we captured traffic on the "local area connection" interface before entering the VPN connection (**S Figure 2**). As a result, all application traffic was accurately recorded, including traffic generated by applications that require a VPN connection.


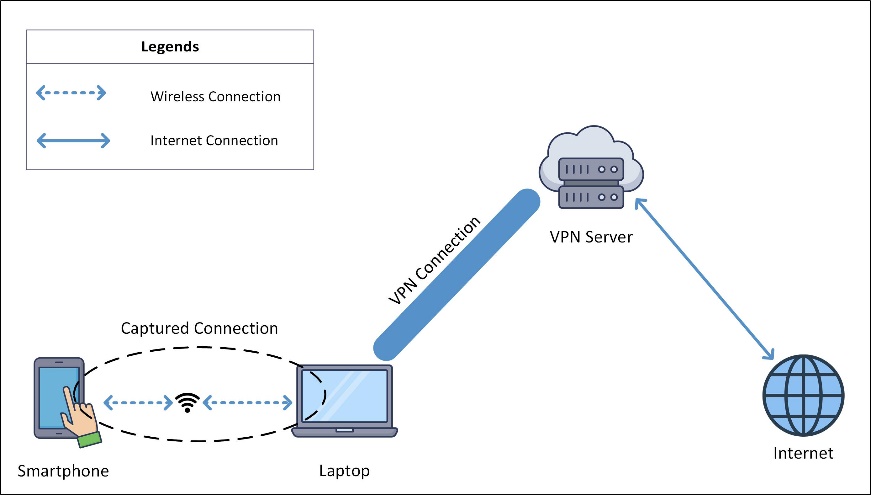


S Figure 2: Capture Setup with VPN.

After collecting traffic data, we separated the target application traffic from the background traffic through a pair-wise comparison of IP addresses and ports captured by Wireshark and PCAPdroid. Specifically, for each trace, we compared each pair of IP addresses and ports captured by Wireshark with all pairs captured by PCAPdroid. Any pairs in Wireshark that did not match a PCAPdroid pair were identified as background traffic and were removed from the Wireshark data. In this way, we eliminated any irrelevant traffic and obtained ground truth without requiring root privileges on mobile phones.

We implemented this method in Python 3 using the Scapy library. The code for this implementation is available in the Supplementary material.

## Traffic Generation

The traffic generation process was carried out by five volunteers from ITC-LAB over six weeks, from October to December 2021. Each volunteer collected traffic from a different network Scenario, which is outlined in **S Table 1.**

S Table 1: Traffic Capture Scenarios Specifications.

| Scenario ID | User | Device | | | Location* | ISP* |
| --- | --- | --- | --- | --- | --- | --- |
|  |  | Vendor | Model | Android version |  |  |
| A | U_1_ | Xiaomi | Note10 Pro | 11 | L_1_, L_2_ | N_1_, N_2_ |
| B | U_2_ | Samsung | A50 | 11 | L_1_, L_3_ | N_1_, N_3_ |
| C | U_3_ | Samsung | A31 | 11 | L_4_ | N_2_, N_4_ |
|  |  |  | Tab A7 Lite | 11 | L_4_ | N_2_, N_4_ |
| D | U_4_ | Samsung | J7 Prime 2 | 9 | L_1_, L_2_, L_5_ | N_1_, N_2_, N_5_ |
| E | U_5_ | Samsung | J7 | 6.0.1 | L_6_ | N_2_, N_6_ |
|  |  |  | A12 | 11 | L_6_ | N_2_, N_6_ |

* L_1_ = ITC Lab L_2_ = District 5, Tehran L_3_ = District 11, Tehran L_4_ =Qom L_5_ = Karaj L_6_ = District 8, Tehran

N_1_ = University of Tehran N_2_ = TCI N_3_ =AsiaTech N_4_ =NTC N_5_ = Shatel N_6_ = MCI

Before commencing the data collection process, the volunteers received training on how to collect traffic. Each volunteer was required to conduct at least three experiments for every application, with each experiment consisting of interacting with a single app on a specific smartphone for 3 to 15 minutes. The volunteers were instructed to use the application as they normally would, to explore its functionalities.

# **dataset specifications**

In this section, the dataset specifications are provided, including the number of packets, bytes, flows, and duration of the capture. The information for each network scenario is presented in **S Table 2**, while **S Table A2** in appendix details this information for each application individually. (The code and related documents are available in the Supplementary Materials.)

S Table 2: Dataset specifications per scenario.

| Scenario ID | No. Packets | No. Bytes (Gigabytes) | No. Bi-Flows^*^ | Capture Duration (hour) |
| --- | --- | --- | --- | --- |
| A | 13,823,338 | 10.04 | 108,370 | 27.95 |
| B | 5,862,397 | 4.08 | 72,279 | 19.42 |
| C | 13,689,836 | 9.60 | 141,957 | 38.37 |
| D | 12,133,832 | 10.15 | 106,652 | 38.54 |
| E | 2,726,719 | 2.04 | 47,044 | 15.07 |
| Total | 48,236,122 | 35.91 | 476,302 | 139.35 |

* The threshold of flows is set to one second.

# **Advantages over available datasets**

The majority of publicly available datasets, as listed in **S Table 3**, have been collected in a single invariant network environment. Only the Appscanner, Cross Market, and CrossNet2021 datasets have been collected in different network scenarios separately. However, these datasets are not without their limitations.

The Appscanner dataset is only available as a statistical feature set, which significantly restricts its practical use. Both the Cross Market and CrossNet2021 datasets, while provided in raw form, suffer from limitations in terms of data volume and the number of apps included.  Their small size makes them unsuitable for methods like deep learning. Furthermore, the limited number of applications can lead to an inaccurate validation, as the number of applications used can influence model accuracy. Notably, the CrossNet2021 dataset includes only 20 Chinese apps, with no representation of international apps such as YouTube.

Our dataset was collected explicitly to address the limitations of existing datasets. By making this dataset publicly available, we hope to facilitate the development of robust and compatible application identification solutions.

S Table 3: Summary of Available Datasets

| Dataset | Mobile Apps Traffic | No. Apps | No. Real Human Users | No. Device | Capture Span | Capture Session Duration | Released Data | No. Network Environments / Scenarios | No. Shared Apps across Scenarios | Shared data volume (Bytes) |
| --- | --- | --- | --- | --- | --- | --- | --- | --- | --- | --- |
| CrossNet2021 [[1](#_ENREF_2)] |  | 20 |  |  |  |  | PCAP files | 2 | 20 | 2.1 GB |
| Appscanner [[2](#_ENREF_3)] | ● | 110 | - (simulation) | 2 |  | 30 min | Feature set | 8 | 65 |  |
| Unicauca [[3](#_ENREF_8)] |  | 78 |  |  | Six days in 2017 |  | Feature set | 1 |  |  |
| Mobilegt [[4](#_ENREF_9)] | ● | 12 | 10 |  | October, 2016 - March, 2017 | 16 min | Feature set | 1 |  |  |
| Andrubis [[5](#_ENREF_10)] | ● | 1M | - (simulation) |  | 2012.06.13 - 2016.03.25 |  | PCAP files | 1 |  |  |
| Mirage [[6](#_ENREF_11)] | ● | 40 | 280 | 1 | May 2017 - May 2019 | 5 - 10 min | Feature set | 1 |  |  |
| Cross Market [[7](#_ENREF_12)] | ● | 400 |  |  | 2017.08.28 - 2017.11.20 |  | PCAP files | 3 | 16 | $\approx$2 MB |
| UTMobileNet2021[[8](#_ENREF_13)] | ● | 16 | - (simulation) | 3 |  |  | PCAP files |  |  |  |
| ITC-Net-Blend-60 | **●** | **60** | **5** | **7** | **October - December 2021** | **3 - 15 min** | PCAP files | **5** | **52** | **35.91 GB** |

*The cells that are left blank are due to the authors not providing any information in those specific areas.

**References**

[1] [dataset] W. Li, X.-Y. Zhang, H. Bao, H. Shi, and Q. Wang, "ProGraph: Robust Network Traffic Identification With Graph Propagation," IEEE/ACM Transactions on Networking, 2022.

[2] [dataset] V. F. Taylor, R. Spolaor, M. Conti, and I. Martinovic, "Robust smartphone app identification via encrypted network traffic analysis," IEEE Transactions on Information Forensics and Security, vol. 13, no. 1, pp. 63-78, 2017.

[3] [dataset] J. S. Rojas, Á. R. Gallón, and J. C. Corrales, "Personalized service degradation policies on OTT applications based on the consumption behavior of users," in Computational Science and Its Applications–ICCSA 2018: 18th International Conference, Melbourne, VIC, Australia, July 2–5, 2018, Proceedings, Part III 18, 2018: Springer, pp. 543-557.

[4] [dataset] R. Wang, Z. Liu, Y. Cai, D. Tang, J. Yang, and Z. Yang, "Benchmark data for mobile app traffic research," in Proceedings of the 15th EAI International Conference on Mobile and Ubiquitous Systems: Computing, Networking and Services, 2018, pp. 402-411.

[5] [dataset] M. Lindorfer, M. Neugschwandtner, L. Weichselbaum, Y. Fratantonio, V. Van Der Veen, and C. Platzer, "Andrubis--1,000,000 apps later: A view on current Android malware behaviors," in 2014 third international workshop on building analysis datasets and gathering experience returns for security (BADGERS), 2014: IEEE, pp. 3-17.

[6] [dataset] G. Aceto, D. Ciuonzo, A. Montieri, V. Persico, and A. Pescapé, "MIRAGE: Mobile-app traffic capture and ground-truth creation," in 2019 4th International Conference on Computing, Communications and Security (ICCCS), 2019: IEEE, pp. 1-8.

[7] [dataset] J. Ren, D. Dubois, and D. Choffnes, "An International View of Privacy Risks for Mobile Apps," ed, 2019.

[8] [dataset] Y. Heng, V. Chandrasekhar, and J. G. Andrews, "UTMobileNetTraffic2021: A Labeled Public Network Traffic Dataset," IEEE Networking Letters, vol. 3, no. 3, pp. 156-160, 2021.

## Appendix

This section contains two tables: **S Table A1** and **S Table A2**.

S Table A1 provides a list of selected applications, while S Table A2 contains detailed specifications for each app.

S Table A1: List of Applications.

| Applications | | | | |
| --- | --- | --- | --- | --- |
| Category | **Name** | **Package Name** | **VPN**  **Requirement** | **Metadata** |
| Books &  Reference | ‎Fidibo | com.fidibo.app | ○ | [Link](https://play.google.com/store/apps/details?id=com.fidibo.app) |
|  | Taghche | ir.mservices.mybook | ○ | [Link](https://cafebazaar.ir/app/ir.mservices.mybook) |
|  | Goodreads | com.goodreads | ● | [Link](https://play.google.com/store/apps/details?id=com.goodreads&hl=en&gl=US) |
| Business | Google Meet | com.google.android.apps.meetings | ○ | [Link](https://play.google.com/store/apps/details?id=com.google.android.apps.meetings&hl=en&gl=US) |
| Communication | Gmail | com.google.android.gm | ○ | [Link](https://play.google.com/store/apps/details?id=com.google.android.gm&hl=en&gl=US) |
|  | Microsoft Outlook | com.microsoft.office.outlook | ○ | [Link](https://play.google.com/store/apps/details?id=com.microsoft.office.outlook&hl=en&gl=US) |
|  | Skype | com.skype.raider | ○ | [Link](https://play.google.com/store/apps/details?id=com.skype.raider&hl=en&gl=US) |
|  | Google Chrome | com.android.chrome | ○ | [Link](https://play.google.com/store/apps/details?id=com.android.chrome&hl=en&gl=US) |
|  | Firefox Browser | org.mozilla.firefox | ○ | [Link](https://play.google.com/store/apps/details?id=org.mozilla.firefox) |
|  | Whatsapp Messenger | com.whatsapp | ○ | [Link](https://play.google.com/store/apps/details?id=com.whatsapp) |
|  | Telegram | org.telegram.messenger | ● | [Link](https://play.google.com/store/apps/details?id=org.telegram.messenger) |
|  | Whatsapp Business | com.whatsapp.w4b | ○ | [Link](https://play.google.com/store/apps/details?id=com.whatsapp.w4b) |
|  | iGap | net.iGap | ○ | [Link](https://play.google.com/store/apps/details?id=net.iGap) |
|  | Soroush Plus Messenger | mobi.mmdt.ottplus | ○ | [Link](https://play.google.com/store/apps/details?id=mobi.mmdt.ottplus) |
|  | Eitaa | ir.eitaa.messenger | ○ | [Link](https://cafebazaar.ir/app/ir.eitaa.messenger) |
| Education | Adobe Connect | air.com.adobe.connectpro | ○ | [Link](https://play.google.com/store/apps/details?id=air.com.adobe.connectpro&hl=en&gl=US) |
|  | Duolingo | com.duolingo | ○ | [Link](https://play.google.com/store/apps/details?id=com.duolingo&hl=en&gl=US) |
|  | Memrise | com.memrise.android.memrisecompanion | ○ | [Link](https://play.google.com/store/apps/details?id=com.memrise.android.memrisecompanion&hl=en&gl=US) |
|  | Coursera | org.coursera.android | ● | [Link](https://play.google.com/store/apps/details?id=org.coursera.android&hl=en&gl=US) |
| Entertainment | Telewebion | net.telewebion | ○ | [Link](https://play.google.com/store/apps/details?id=net.telewebion) |
|  | Youtube | com.google.android.youtube | ● | [Link](https://play.google.com/store/apps/details?id=com.google.android.youtube&hl=en&gl=US) |
|  | Aparat | com.aparat | ○ | [Link](https://cafebazaar.ir/app/com.aparat) |
|  | Filimo | com.aparat.filimo | ○ | [Link](https://cafebazaar.ir/app/com.aparat.filimo) |
| Finance | Ewano | com.ebcom.ewano | ○ | [Link](https://play.google.com/store/apps/details?id=com.ebcom.ewano) |
|  | AP - Asan Pardakht | com.sibche.aspardproject.app | ○ | [Link](https://play.google.com/store/apps/details?id=com.sibche.aspardproject.app) |
| Game | Clash of Clans | com.supercell.clashofclans | ○ | [Link](https://play.google.com/store/apps/details?id=com.supercell.clashofclans&hl=en&gl=US) |
|  | Football Strike | com.miniclip.footballstrike | ○ | [Link](https://play.google.com/store/apps/details?id=com.miniclip.footballstrike&hl=en&gl=US) |
|  | Mencherz | com.incyteltech.mencherz | ○ | [Link](https://play.google.com/store/apps/details?id=com.incyteltech.mencherz&hl=en&gl=US) |
|  | Quiz Of Kings | co.palang.QuizOfKingss | ○ | [Link](https://play.google.com/store/apps/details?id=co.palang.QuizOfKingss&hl=en&gl=US) |
|  | Mafioso | com.herocraft.game.mafioso.gangster.paradise.pvp | ○ | [Link](https://play.google.com/store/apps/details?id=com.herocraft.game.mafioso.gangster.paradise.pvp) |
| Lifestyle | Pinterest | com.pinterest | ○ | [Link](https://play.google.com/store/apps/details?id=com.pinterest) |
| Maps & navigation | Snapp | cab.snapp.passenger.play | ○ | [Link](https://play.google.com/store/apps/details?id=cab.snapp.passenger.play) |
|  | Balad | com.baladmaps | ○ | [Link](https://play.google.com/store/apps/details?id=com.baladmaps) |
|  | Neshan | org.rajman.neshan.traffic.tehran.navigator | ○ | [Link](https://play.google.com/store/apps/details?id=org.rajman.neshan.traffic.tehran.navigator) |
|  | Tapsi | taxi.tapsi.passenger | ○ | [Link](https://play.google.com/store/apps/details?id=taxi.tapsi.passenger) |
|  | Google Maps | com.google.android.apps.maps | ○ | [Link](https://play.google.com/store/apps/details?id=com.google.android.apps.maps&hl=en&gl=US) |
|  | Waze | com.waze | ● | [Link](https://play.google.com/store/apps/details?id=com.waze&hl=en&gl=US) |
| Music & Audio | Radio Javan | com.radiojavan.androidradio | ○ | [Link](https://play.google.com/store/apps/details?id=com.radiojavan.androidradio) |
|  | Castbox | fm.castbox.audiobook.radio.podcast | ○ | [Link](https://play.google.com/store/apps/details?id=fm.castbox.audiobook.radio.podcast&hl=en&gl=US) |
|  | Spotify | com.spotify.music | ● | [Link](https://play.google.com/store/apps/details?id=com.spotify.music&hl=en&gl=US) |
| Photography | Facelab | com.lyrebirdstudio.facelab | ○ | [Link](https://play.google.com/store/apps/details?id=com.lyrebirdstudio.facelab) |
|  | ToonMe | com.vicman.toonmeapp | ○ | [Link](https://play.google.com/store/apps/details?id=com.vicman.toonmeapp) |
| Productivity | Dropbox | com.dropbox.android | ○ | [Link](https://play.google.com/store/apps/details?id=com.dropbox.android&hl=en&gl=US) |
|  | OneDrive | com.microsoft.skydrive | ○ | [Link](https://play.google.com/store/apps/details?id=com.microsoft.skydrive&hl=en&gl=US) |
| Shopping | Divar | ir.divar | ○ | [Link](https://play.google.com/store/apps/details?id=ir.divar) |
| Shopping | Digikala | com.digikala.diagon | ○ | [Link](https://play.google.com/store/apps/details?id=com.digikala.diagon) |
|  | Shaypur | com.sheypoor.mobile | ○ | [Link](https://play.google.com/store/apps/details?id=com.sheypoor.mobile) |
|  | Torob | ir.torob | ○ | [Link](https://play.google.com/store/apps/details?id=ir.torob) |
| Social | LinkedIn | com.linkedin.android | ○ | [Link](https://play.google.com/store/apps/details?id=com.linkedin.android&hl=en&gl=US) |
|  | Snapchat | com.snapchat.android | ● | [Link](https://play.google.com/store/apps/details?id=com.snapchat.android) |
|  | Instagram | com.instagram.android | ○ | [Link](https://play.google.com/store/apps/details?id=com.instagram.android) |
|  | Likee | video.like | ● | [Link](https://play.google.com/store/apps/details?id=video.like) |
|  | Facebook lite | com.facebook.lite | ● | [Link](https://play.google.com/store/apps/details?id=com.facebook.lite) |
|  | Clubhouse | com.clubhouse.app | ○ | [Link](https://play.google.com/store/apps/details?id=com.clubhouse.app) |
|  | Discord | com.discord | ○ | [Link](https://play.google.com/store/apps/details?id=com.discord&hl=en&gl=US) |
|  | Twitter | com.twitter.android&hl | ● | [Link](https://play.google.com/store/apps/details?id=com.twitter.android&hl=en&gl=US) |
| Android app market | Google Play Store | com.google.vending | ○ | Link |
|  | Myket | ir.mservices.market | ○ | Link |
|  | Bazaar | com.farstitel.bazaar | ○ | Link |
| Tools | InSave | instagram.status.hd.images.video.downloader | ○ | [Link](https://play.google.com/store/apps/details?id=instagram.status.hd.images.video.downloader) |

S Table A2: Dataset specifications per app.

| App | No. Packets | No. Bytes (Megabytes) | No. Bi-Flows | Capture Duration (hour) |
| --- | --- | --- | --- | --- |
| AP - Asan Pardakht | 556,407 | 399 | 8,753 | 2.29 |
| Adobe Connect | 409,437 | 169 | 612 | 1.79 |
| Aparat | 2,029,423 | 1,794 | 4,125 | 2.27 |
| Balad | 484,595 | 375 | 4,871 | 2.22 |
| Bazaar | 1,641,324 | 1,401 | 5,128 | 2.34 |
| Castbox | 3,082,176 | 2,311 | 18,963 | 2.33 |
| Clash of Clans | 748,481 | 569 | 5,871 | 2.54 |
| Clubhouse | 903,104 | 237 | 6,537 | 2.20 |
| Coursera | 594,895 | 423 | 6,552 | 2.16 |
| Digikala | 500,848 | 367 | 8,740 | 2.60 |
| Discord | 680,814 | 491 | 8,569 | 2.64 |
| Divar | 169,584 | 108 | 4,261 | 2.08 |
| Dropbox | 455,980 | 351 | 3,753 | 1.76 |
| Duolingo | 497,763 | 366 | 7,671 | 2.34 |
| Eitaa | 308,086 | 233 | 3,010 | 2.13 |
| Ewano | 543,186 | 437 | 3,451 | 1.97 |
| Facebook Lite | 932,373 | 710 | 4,861 | 2.44 |
| Facelab | 398,880 | 255 | 14,188 | 2.14 |
| Fidibo | 523,916 | 440 | 6,251 | 2.07 |
| Filimo | 2,031,333 | 1,908 | 4,549 | 2.31 |
| Firefox Browser | 797,195 | 571 | 14,196 | 2.27 |
| Football Strike | 220,144 | 128 | 10,139 | 2.42 |
| Gmail | 272,916 | 182 | 4,963 | 2.22 |
| Goodreads | 306,978 | 170 | 10,388 | 2.06 |
| Google Chrome | 1,029,370 | 864 | 19,816 | 2.55 |
| Google Maps | 533,560 | 388 | 4,155 | 2.31 |
| Google Meet | 887,544 | 423 | 6,823 | 2.25 |
| Google Play Store | 915,959 | 800 | 3,042 | 2.47 |
| iGap | 743,193 | 587 | 5,516 | 2.67 |
| InSave | 879,991 | 668 | 10,680 | 2.23 |
| Instagram | 1,896,262 | 1,753 | 13,150 | 3.01 |
| Likee | 2,400,719 | 1,618 | 35,462 | 2.40 |
| LinkedIn | 427,895 | 309 | 3,825 | 2.52 |
| Mafioso | 523,166 | 434 | 7,554 | 3.19 |
| Memrise | 135,317 | 95 | 5,802 | 2.35 |
| Mencherz | 41,471 | 11 | 4,426 | 2.88 |
| Microsoft Outlook | 127,217 | 65 | 6,914 | 1.83 |
| Myket | 1,818,709 | 1,526 | 8,884 | 2.13 |
| Neshan | 347,192 | 186 | 11,785 | 2.05 |
| OneDrive | 198,708 | 131 | 4,503 | 2.07 |
| Pinterest | 1,623,021 | 1,299 | 6,285 | 2.13 |
| Quiz Of Kings | 167,978 | 94 | 8,991 | 2.79 |
| Radio Javan | 2,255,378 | 1,903 | 14,564 | 3.00 |
| Shaypur | 171,050 | 84 | 8,138 | 2.14 |
| Skype | 838,077 | 392 | 8,542 | 2.98 |
| Snapchat | 1,403,567 | 1,058 | 13,871 | 2.59 |
| Snapp | 488,416 | 349 | 9,273 | 2.58 |
| Soroush Plus Messenger | 1,392,792 | 1,190 | 11,108 | 2.37 |
| Spotify | 882,907 | 587 | 6,843 | 2.45 |
| Taghche | 469,307 | 341 | 8,527 | 2.45 |
| Tapsi | 150,856 | 98 | 3,823 | 1.93 |
| Telegram | 707,839 | 498 | 7,519 | 2.78 |
| Telewebion | 1,693,306 | 1,531 | 4,574 | 1.86 |
| ToonMe | 612,690 | 459 | 14,950 | 2.31 |
| Torob | 861,487 | 699 | 5,442 | 2.27 |
| Twitter | 453,923 | 312 | 5,434 | 2.08 |
| Waze | 310,158 | 210 | 4,193 | 1.65 |
| Whatsapp Business | 179,550 | 143 | 1,504 | 1.13 |
| Whatsapp Messenger | 597,216 | 462 | 3,550 | 2.62 |
| Youtube | 980,493 | 811 | 6,432 | 2.68 |

1. Cafe Bazaar. Available online: [https://cafebazaar.ir/app/(](https://cafebazaar.ir/app/)accessed on 24 February 2024). [↑](#footnote-ref-1)
2. Myket. Available online: <https://myket.ir/>(accessed on 24 February 2024). [↑](#footnote-ref-2)
3. PCAPdroid. Available online: [https://github.com/emanuele-f/PCAPdroid/](https://github.com/emanuele-f/PCAPdroid/%20) (accessed on 21 May 2023). [↑](#footnote-ref-3)
4. ProtonVPN. Available online: <https://protonvpn.com/>(accessed on 21 May 2023). [↑](#footnote-ref-4)
